# Manual for primary health care on Basic Occupational Health Services. Encouraging publication from India, focused on informal occupations

**DOI:** 10.1186/s12930-017-0032-8

**Published:** 2017-01-19

**Authors:** Frank van Dijk, Peter Buijs

**Affiliations:** 1 Learning and Developing Occupational Health (LDOH) Foundation, Speelkamp 28, 3831 PE Leusden, The Netherlands; 2 Department of Public and Occupational Health, EMGO+ Institute for Health and Care Research, VU University Medical Center Amsterdam, Amsterdam, The Netherlands

**Keywords:** Primary health care, Family physician, Community health care, Occupational health, Workers’ health, Informal worker, Occupational diseases, Prevention, Return to work

## Abstract

**Objective:**

To discuss a new book from India intended to inform and educate primary health care professionals on workers’ health problems, with the aim to encourage new initiatives.

**Study design:**

The book is considered against the background of international developments and evaluated on the usefulness for practice and policy development.

**Results:**

The publication focuses on the 90% of the workers in India working informal, without a contract or social security, and often exposed to poor working conditions. It is the final aim of the book to prioritize care for those at the highest risk. For informal workers specialized occupational health services are absent. Therefore, primary health care might take care of basic facilities on workers’ health, when educated and adequately supported by online information, occupational health experts and clinical referral services. Such new developments started as well in other countries such as China, Thailand, Iran and Indonesia, encouraged by WHO, WONCA (family physicians), ILO and ICOH (occupational health experts). In the book working conditions are described in 22 branches of economic activities in India with many informal workers like agriculture, leather and tanning industry, oil mills and street vendors. Next, associated health complaints and occupational diseases are explained. This information is relevant for family physicians to be able to recognize work-relatedness of health complaints and diseases. Numerous diseases can be work related such as asthma, depressive disorders, dermatitis, a variety of musculoskeletal disorders, hearing impairment, cancer of many organs, various infectious and neurological diseases. Diagnosis, treatment and prevention can be improved in primary health care, as well as advising in return to work activities. More detailed information on specific occupational or work-related diseases is given in clinical chapters. Comments are given to improve the usefulness in supporting new practices and policies.

**Conclusion:**

This book from India fits well in worldwide developments promoting the integration of forms of workers’ health care in primary health care.

## Background

Like many other countries, India has a serious occupational health service coverage problem. Therefore, the Indian Association of Occupational Health published a book (391 pages) on *Basic Occupational Health Services for the informal Industry* [1]. The book and associated project can be appreciated within the WHO strategy of Universal Health Coverage, focusing on workers’ health [2].

The *Basic Occupational Health Services (BOHS)* strategy of World Health Organization (WHO), International Labor Organization (ILO) [3] and International Commission on Occupational Health (ICOH) [4] is the chosen fundament of the project. Primary health care is considered as a first level of “*workers’ health* care” for the about 85% of 3.1 billion workers worldwide who are not supported by expert-based Occupational Health Services [5]. As is stated in the book “In the Indian context the coverage of such services is minimal for the unorganized sector (informal workers) and hardly satisfying the expectations of the workers regarding access to interventions and protective measures through basic occupational health services.” There is a vast inequality in the practice of prevention of occupational diseases and in the development of healthy workplaces in India. In the vision of the authors, basic care for workers’ health should be an integral part of a primary health care. Provision of occupational health care could be prioritized “for those in needs and at greatest risk, through the financing mechanisms existing for primary health care”.

Well-supported primary or community health care can deliver basic care for workers including a significant level of surveillance of the work environment, advises for preventive measures, education, health surveillance for early detection of occupational diseases, first aid and return to work support [6, 7]. The WHO The Hague Conference [8] and the common Statement and Pledge of World Organization of Family Doctors (WONCA) and ICOH in Lisbon in 2014 [9] are cited in the book as signs of a new global policy. Correspondingly, education and training of primary care providers is the main objective of this Indian project, sensitizing the professionals on health risks in 22 informal economic activities and occupations.

The ambitious ultimate target of the project is the improvement of health and safety of about 450 million workers in India. More than 90% of these work in the informal sector, often in poor conditions and consequently with a high risk of occupational diseases and accidents. Unfortunately, statistics are not easily available. The authors state that hardly any informal worker receives professional support in health and safety.

## The manual, aim and objectives

The book, written by a long list of experts, has as a main aim the education and training of primary care providers. Primary health care may contribute to a better health care for workers, but in practice physicians and staff are often not interested in the patients’ occupation, caused by “a lack of awareness and knowledge about work-related hazards”.

A first objective is sensitizing the professionals on the vital relation that exist between work of the patients and their health. In the first two sections of the book concepts and legislation on occupational health are introduced, and basic thoughts about hazards and risks, safety principles and health surveillance. Instruments such as occupational history taking, occupational hygiene tools and monitoring are shortly described.

A second objective is to inform primary health care about a wide range of concrete risks at workplaces where most informal workers have their job. The authors do so by presenting profiles of no less than 22 informal economic activities which exist mainly in rural areas.

Finally, to support finding medical solutions for patients, clinical perspectives are offered for 10 categories of diseases.

## Risks in informal economic activities and occupations

In 22 chapters the profiles of informal economic activities and occupations are presented. First the type of activity is portrayed informing about economic data, regional distribution, numbers of workers and employment conditions. Next, work processes and working conditions are described in detail and information on hygienic conditions (toilets, drinking water). Finally, specific work-related and occupational diseases are mentioned, completed with causative sources at work. References are added for additional clinical information presented in the final section of the book.

Such information can support the family physician, nurse or community health worker while identifying the cause of a suspected occupational or work-related disease such as in diagnosing asthma, contact eczema, tuberculosis, epicondylitis, depression or bladder cancer. Clearly, the risk of missing the right diagnosis is high without basic awareness and information regarding specific risks at the workplace.

Preventive activities during office hours are encouraged as well. As an illustration, in the chapter on *Agriculture* you can find that almost 300,000 Indian farmers, especially cash crop farmers, committed suicide in the period 1995–2013 caused by fundamental work-related problems. Primary health care professionals are stimulated to have an open eye for this risk in contacts with farmers/patients with depressive mood complaints or anxiety problems. Information on common risks in a branch can guide professionals during a work site visit. So, when visiting a company in the *Leather and tanning industry* it is important to know that chromium exposure can be a risk for getting lung, skin and nasal cancer. Other chemicals may cause bladder or kidney cancer, pancreatic and testicular cancer.

In the chapter on *Saw mills* attention is given to safety risks and noise, as could be expected, but also to dermatitis and respiratory diseases caused by wood dust, fungal spores and chemicals. In *Oil mills* workers can be exposed to extreme heat leading to heat exhaustion and heat stroke. No less than 2.5 million S*treet vendors* are working in the city of Mumbai alone. Work characteristics and related health problems are explicated in the corresponding chapter.

An innovative aspect of the book is the profiling of branches and occupations hardly described in the scientific literature. An example is *Work in salt pans*. In PubMed you can find one scientific article describing work processes but no articles on work and health topics.

## Clinical perspectives

After a generic description, information is given on pathogenesis, symptoms, general risk factors, diagnosis, tests and treatment. A number of chapters open with an adequate introduction on work-related diseases. The chapters on *Respiratory System Conditions* and *Skin Health Conditions* contain informative subchapters on various work-related and occupational diseases. In the chapter on *Occupational Neoplastic Disease* the reader is informed about potential occupational diseases such as skin cancer, melanoma, bladder cancer, leukemia, lung cancer and mesothelioma. Interesting paragraphs on toxic encephalopathy, ‘distress as a result of poverty’, suicide and depression can be found in the chapter on *Neurological and Behavioural Conditions*. Refreshing is the attention for rehabilitation when workers have a cervicobrachial syndrome as can be found in the chapter on *Musculoskeletal Disorders*.

Surprisingly, common diagnoses in high-income but increasingly also in lower-income countries such as adjustment disorders (nervous exhaustion) and burnout, are not discussed. Similarly only limited attention is given to widespread work-related diseases as chronic low back pain and epicondylitis.

The authors may consider giving more epidemiologic data on occupational and work-related diseases (when available). The presentation of more good practices for the detection, diagnosis and surveillance of occupational and work-related diseases, and for prevention and rehabilitation, could stimulate the realization in primary care.

## Support for a new practice and policy

The publication can play a significant role in supporting primary health care professionals with learning material for meetings and courses, contributing to a better quality of health care practice and positive changes for the health of informal workers.

The book is not developed as a ‘how to do’ book with clear procedures for practice such as on how to perform an occupational history, how to assess occupational asthma, or how to reduce asbestos exposure in a garage. An idea could be to write a separate ‘practice book’ including tailor-made concepts and visualizations for primary health care, good practices and references to appropriate online sources and Apps. Especially effective workplace solutions and other preventive measures are missing in this edition.

Readability is important to stimulate the use of the book. In this perspective, information overload can be reduced by deleting detailed information on hospital-based diagnosis and therapy. Subchapters on not work-related diseases might not be needed. A needs assessment might be considered to strengthen the orientation of the book on primary and community health care.

The inevitability to establish a supportive infrastructure including expert support and referral options for primary care could get more attention, following WHO policies. Consequently, the number of occupational safety and health experts has to increase rapidly not only to take care for the larger industries, but also to support primary health care.

The capability of primary or community health care to perform good basic occupational health care as a well-organized, mostly government supported form of care can be observed in countries such as China, Thailand, Indonesia and Iran [10–13]. Such investments are sometimes motivated by the economic costs of work-related injuries and diseases that vary between 1.8 and 6.0% of Gross Domestic Product (GDP) in country estimates [14]. However, one may also argue, citing the words of the former Secretary General of the United Nations Kofi Annan in 1997, that “health and safety at work is not only sound economic policy, it is a basic human right”.

## Quality of the information

Details can be challenged, surely in a first edition of a comprehensive book. As an illustration, in the chapter on bladder cancer no preventive measures can be found to reduce industrial exposure, and urine cytology as an instrument for a workers’ health surveillance program can be discussed more in detail, leading to a recommendation for a health surveillance program. For the next edition, expert referees can screen the text for mistakes and missing parts.

The book has no references per chapter. However, the reader needs references to verify the validity of the content and to know where to find tools or sources for further reading. On the other hand, such a comprehensive book creates a huge amount of quickly outdated references when not well organized. A solution could be restricting references mainly to recent systematic reviews and evidence-based guidelines.

## Next steps

Next steps are planned. First seminars are hold for family physicians focusing on a few informal economic activities. Concrete plans are in development for telecasting modules to primary care centers, including translated versions in local language for paramedics and local health workers. As the so called “Blind Spot within health care for work” is wide spread, these initiatives are interesting for other countries [15]. The use of modern online and blended education and information technics is envisaged in the book. This part might be elaborated in the future. What about massive open online courses (MOOCs), and the application of apps for professionals, workers and companies [16, 17], YouTube videos and ‘serious games’? Of course adequate funding is needed.

## International collaboration

This inspiring book from India invites us to intensify our national efforts and the international collaboration that is still in the earliest phase. International agencies could collaborate more intensively and effectively. Long-term funding is essential to make significant progress as a number of national initiatives are waiting for support, evaluation, sharing and up scaling.

## Conclusions

This book from India aims at informing and educating primary health care professionals about working conditions of informal workers and related occupational and work-related diseases. Recognition, diagnosis, treatment and prevention can be improved in daily practice. The initiative fits well in worldwide developments in which educated and supported primary health care is testing and integrating new basic forms of workers’ health care, “to the benefit of all workers and their families” (WONCA-ICOH Pledge) [9].
